# Association between salivary serotonin and the social sharing of happiness

**DOI:** 10.1371/journal.pone.0180391

**Published:** 2017-07-06

**Authors:** Masahiro Matsunaga, Keiko Ishii, Yohsuke Ohtsubo, Yasuki Noguchi, Misaki Ochi, Hidenori Yamasue

**Affiliations:** 1 Department of Health and Psychosocial Medicine, Aichi Medical University School of Medicine, Nagakute, Aichi, Japan; 2 Department of Psychology, Graduate School of Humanities, Kobe University Faculty of Letters, Kobe, Hyogo, Japan; 3 Department of Psychiatry, Hamamatsu University School of Medicine, Hamamatsu, Shizuoka, Japan; Chiba Daigaku, JAPAN

## Abstract

Although human saliva contains the monoamine serotonin, which plays a key role in the modulation of emotional states, the association between salivary serotonin and empathic ability remains unclear. In order to elucidate the associations between salivary serotonin levels, trait empathy, and the sharing effect of emotions (i.e., sharing emotional experiences with others), we performed a vignette-based study. Participants were asked to evaluate their happiness when they experience several hypothetical life events, whereby we manipulated the valence of the imagined event (positive, neutral, or negative), as well as the presence of a friend (absent, positive, or negative). Results indicated that the presence of a happy friend significantly enhanced participants’ happiness. Correlation analysis demonstrated that salivary serotonin levels were negatively correlated with happiness when both the self and friend conditions were positive. Correlation analysis also indicated a negative relationship between salivary serotonin levels and trait empathy (particularly in perspective taking), which was measured by the Interpersonal Reactivity Index. Furthermore, an exploratory multiple regression analysis suggested that mothers’ attention during childhood predicted salivary serotonin levels. Our findings indicate that empathic abilities and the social sharing of happiness decreases as a function of salivary serotonin levels.

## Introduction

Empathy is a fundamental human feature and allows us to share and understand each other’s internal states, beliefs, and intentions [1–5]. Previous studies have indicated that our own emotional states are strongly influenced by the presence of others [4–6]. For instance, one’s positive feelings can be elevated when viewing positive stimuli with a friend, compared to viewing alone [6]. Research has shown that positive feelings are contagious, as the presence of a happy person enhances others’ happiness [7,8]. Conversely, negative emotions are also subject to the influence of the presence of others. For example, previous studies have indicated that interpersonal contact alleviates negative feelings induced by aversive stimuli [9]. Such interpersonal emotional processes are termed the “social sharing of emotions” [10].

It is well known that there are individual differences in empathic ability, which is associated with the susceptibility of being influenced by both the positive and negative emotional states of others [11–13]. Previous studies have also indicated an association between trait empathy and helping behaviors, such as caregiving intentions toward infant crying [14]. Although biological mechanisms underlying such individual differences in empathic abilities are not yet fully understood, one possible candidate may be the actions of the serotonin (5-hydroxytryptamine; 5-HT) system, which plays a key role in the modulation of emotional states [15,16]. Previous studies have demonstrated that administration of serotonin 2A receptor agonists (lysergic acid diethylamide or psilocybin) to healthy participants can influence empathic abilities [15,16]. For example, a previous study demonstrated that administration of lysergic acid diethylamide to healthy participants attenuated the ability to correctly infer the mental state of others, as assessed by the Multifaceted Empathy Test [15]. These findings suggest that serotonergic activities might strongly influence the sharing effect of emotions.

Human saliva contains various hormones, including steroids, peptides, and amines [17]. Researchers have indicated associations between levels of several salivary hormones, such as cortisol and cytokines, and psychological measures, such as subjective stress [18–20]. Serotonin is also detectable in human saliva [21]. It is possible that salivary serotonin is derived from the blood, as it has not yet been confirmed that the serotonergic system directly projects from the brain to salivary glands [22]. A previous study indicated a positive association between recovery rate in terms of depressive symptoms, and an increase in circadian amplitude of salivary serotonin secretion, in patients with depression after clinical treatment (fluoxetine administration) [21]. This suggests that salivary serotonin may be useful for the assessment of serotonergic functions. However, to the best of our knowledge, it is still unknown whether salivary serotonin levels are associated with empathic abilities.

Another line of research implicates the involvement of childhood environment in individual differences in serotonin functions [23–26]. In animal studies, it has been demonstrated that neonatal maternal separation can influence central serotonin receptor expressions and pain-induced serotonin responses [24,26]. It has also been observed in humans that childhood maltreatment decreases central serotonergic neurotransmission in adults [25], and self-rated childhood emotional neglect has been shown to relate negatively to monoamine levels (such as the serotonin metabolite 5-hydroxyindoleacetic acid, 5-HIAA), in cerebrospinal fluid [23]. Researchers have also reported that childhood adversity, such as childhood maltreatment, is associated with impaired empathy [27,28]. Based on these previous findings, it is suggested that childhood environment affects serotonergic functions and is effective in predicting salivary serotonin levels.

In this study, in order to elucidate associations between salivary serotonin levels, trait empathy, and the emotion sharing effect, we conducted a vignette-based study and saliva collection. This formed part of a wider study involving the administration of various measures of individual differences (e.g., a measure of trait empathy). The participants were asked to evaluate their happiness if they were to experience several hypothetical life events, with or without a friend present. We investigated whether happiness was altered by the hypothetical presence of their friend (i.e., the sharing effect of emotions), and whether this sharing effect was correlated with salivary serotonin levels and trait empathy. In addition, the participants were asked to evaluate their parents’ attention toward them during childhood. This information was collected in an attempt to determine whether childhood environment is associated with salivary serotonin levels and empathic abilities.

## Materials and methods

### Participants

We recruited 213 healthy male and female volunteers (age range: 18–25 years; mean age: 19.26 years; 100 males, 112 females, one unreported) following the study’s approval by the Experimental Research Ethics Committee at the Graduate School of Humanities, Kobe University (approval number: 2014–10). All participants provided written informed consent in accordance with the Declaration of Helsinki. Participants were recruited through a psychology subject pool and all were Japanese undergraduate students at Kobe University. The present study was conducted as a part of a half-day experiment, including several questionnaires on the self, emotion, cognition, and interpersonal behaviors. Participants were paid 4000 yen (approximately 40 USD). One participant felt ill and withdrew during the session. No participant was taking psychotropic drugs that affect serotonin systems, such as selective serotonin reuptake inhibitors (SSRIs), during the study. To eliminate possible confounding effects of gender, age, and psychiatric diseases [29,30], we focused on university students. Although they do not comprise a representative sample of the healthy, young Japanese population, they exhibited reasonable levels of variance in happiness, empathy, salivary serotonin, and childhood parental attention. Therefore, it was deemed that the correlation-based analyses were unlikely to be affected by the range restriction problem. The questionnaires and saliva collection were conducted at Kobe University, and the saliva serotonin assaying was conducted at Aichi Medical University.

We tried to collect as much data as possible under research funding constraints. Statistical power analysis was conducted using G*Power version 3.1.9.2 [31]. We assumed that the effect size of this study would be equivalent to that observed in a study by Ito et al. [32] that found a significant correlation between psychological measures and salivary levels of melatonin, a metabolite of serotonin. *A priori* power analysis estimated the necessary sample size for this study as *n* = 191 (two-tailed t tests; effect size = 0.2; alpha error = 0.05; 1−beta error = 0.8).

The mean body mass index (BMI), which is related to several mental health issues [33], was 20.6 kg/m^2^ (BMI range: 12.59–32.74 kg/m^2^). The number of participants in four BMI categories were as follows: underweight (< 18.5 kg/m^2^), *n* = 30; normal weight (18.5–24.9 kg/m^2^), *n* = 171; overweight (25–29.9 kg/m^2^), *n* = 8; and class I obese (30–34.9 kg/m^2^), *n* = 2. The BMI of two participants could not be calculated due to the omission of questionnaires. Thus, most participants exhibited a normal weight range, which was not linked to mental dysfunction. No participants smoked, although 27 participants consumed alcohol.

#### Evaluation of trait empathy

A Japanese version of the Interpersonal Reactivity Index (IRI) [34] was administered to assess participants’ empathic traits. The questionnaire consists of 28-items answered on a 5-point Likert scale ranging from *does not describe me well* to *describes me very well*. The measure has four subscales (perspective taking, fantasy, empathic concern, and personal distress), each made up of seven different items. Cronbach’s alpha for “perspective taking” was 0.63, “fantasy” was 0.55, “empathic concern” was 0.36, and for “personal distress” was 0.73, in the present study. Given that the reliability of empathic concern was low (cf. Cronbach’s alpha was slightly low, 0.67 in Aketa’s original report), we excluded it from the subsequent analyses. There were 15 participants who missed the final page of the questionnaire. Their empathy scores were computed based on the completed items. One participant's perspective taking score was not computed, as there were too many missing values. The data from this participant was also omitted from the analyses involving the perspective taking score. Overall, Cronbach’s alpha of the IRI in the current study was low. However, in the original study, this measure was also not very high (Cronbach’s alpha for “perspective taking” was 0.71, “fantasy” was 0.85, and “personal distress” was 0.67 in Aketa’s original report). Thus, it is possible that there were some problems in several items in the Japanese version of the IRI. Based on this evidence, a newer version was created in 2017 [35]. However, at the time this research was conducted, the improved version was not available. Thus, the existing (best) Japanese version, which had been examined for reliability and validity, was used in the current experiment.

#### Life event vignette

Based on our previous psychological study, we created a vignette to assess the emotion sharing effect [36]. The hypothetical event had three components: the occasion, the self-outcome, and the friend’s situation. Participants were instructed to think of a same-sex friend. We selected one occasion (When you are striving to achieve a personal goal). In this occasion, the valence of the outcome that participants imagined to experience by themselves (positive, neutral, or negative) and the situation of their friend (absent, positive, or negative) were manipulated in order to examine what type of empathy (positive empathy or negative empathy) was associated with salivary serotonin levels. Therefore, the participants were asked to evaluate their happiness on a 7-point Likert scale (1: *extremely unhappy*; 2: *very unhappy*; 3: *a little unhappy*; 4: *neither*; 5: *a little happy*; 6: *very happy*; 7: *extremely happy*) when they encountered the following nine situations: “You achieved the goal (your friend is irrelevant to this event)” (self-positive/absent condition); “Both you and your friend achieved the goal” (self-positive/friend-positive condition); “You achieved the goal, but your friend failed to achieve his/her goal” (self-positive/friend-negative condition); “You are still on the way to achieving the goal (your friend is irrelevant to this event)” (self-neutral/absent condition); “You are still on the way to achieving the goal, but your friend achieved his/her goal” (self-neutral/friend-positive condition); “You are still on the way to achieving the goal, but your friend failed to achieve his/her goal” (self-neutral/friend-negative condition); “You failed to achieve the goal (your friend is irrelevant to this event)” (self-negative/absent condition); “You failed to achieve the goal, but your friend achieved his//her goal” (self-negative/friend-positive condition); and “Both you and your friend failed to achieve the goal” (self-negative/friend-negative condition).

#### Evaluation of parents’ attention during childhood

In order to reveal the association between childhood environment and salivary serotonin levels, the participants were asked to evaluate their parents’ attention toward them during childhood on a 6-point Likert scale (1: *extremely inattentive*; 2: *inattentive*; 3: *a little inattentive*; 4: *a little attentive*; 5: *attentive*; 6: *extremely attentive*), with regard to the following two questions: “When you were a child, how much did your father direct attention toward you?” and “When you were a child, how much did your mother direct attention toward you?” Although the reliability and validity of the evaluation were not examined in the current study, similar retrospective-report measures of early family environments were successfully used in previous studies [37,38].

#### Measurement of salivary serotonin

Considering the circadian rhythm of salivary serotonin in healthy participants [21], saliva samples were collected between 11:00 a.m. and 1:00 p.m. The participants did not eat at least 1 hour before saliva collection, and rinsed their mouth with water at least 30 minutes before collecting saliva to avoid sample dilution. After mouth wash, they were instructed not to drink anything. Then, the experimenter gave participants a saliva collection device (Salimetrics, LLC, State College, PA) and explained how to use it. The saliva samples were temporarily stored at Kobe University at −20°C, and then stored at Aichi Medical University at −80°C until analysis. The samples were centrifuged at 3,500 rpm for 15 min before analysis. The concentration of serotonin in saliva was determined by an enzyme-linked immunosorbent assay (ELISA) using a serotonin ELISA Kit (Enzo Life Sciences, Inc., Farmingdale, NY). The intra-assay coefficient of variation for each sample was 4.2–11.0%, and inter-assay coefficient of variation for each sample was 12.7–16.2%. The sensitivity was 0.293 ng/ml (range 0.49–500 ng/ml). Concerning the accuracy of the serotonin assay, the cross reactivities for a number of related compounds were as follows: N-Acetyl serotonin: 17%, 5-Hydroxy-L-tryptophan: 0.4%, Tryptamine: 0.1%, 5-Hydroxyindoleacetic acid: 0.03%, Melatonin: 0.01%, Tyramine: < 0.004%, and Tryptophan: < 0.004%. Salivary serotonin of four participants was not measured due to loss of saliva samples. Further, serotonin of 26 participants was not measured due to the serotonin being below the detection limit. Therefore, we analyzed the association between salivary serotonin and several psychological measures in 183 participants. All samples were analyzed in duplicate with operators blind to any psychological data.

### Statistical analyses

All data analyses were conducted using SPSS version 18 (International Business Machines Corporation (IBM), Armonk, NY). Study variables were first compared between males and females by Student’s *t* tests. The rating scores for happiness in the questionnaire were compared using a two-factor [self-valence (positive, neutral, or negative) and situation of a friend (absent, positive, or negative)] analysis of variance (ANOVA), followed by a Bonferroni’s multiple comparisons test. In order to reveal the associations between salivary serotonin, trait empathy, and happiness in the vignette study, Pearson’s correlation coefficients were computed. Furthermore, in order to remove the influences of several confounding factors, such as age, sex, BMI, and drinking habits, from the effects of salivary serotonin on trait empathy or happiness, we used the following multiple regression model:

$$Y=\beta_{0}+\beta_{1}A+\beta_{2}B+\beta_{3}C+\beta_{4}D+\beta_{5}E+\varepsilon$$

In the above formula, “Y” is a dependent variable (trait empathy or happiness); “A” is a matrix of variables to control for the salivary serotonin concentration; “B” is a matrix of variables to control for sex (B = 1 if the subject is male and B = 2 if the subject is female); “C” is a matrix of variables to control for age; “D” is a matrix of variables to control for BMI; “E” is a matrix of variables to control for drinking habits (E = 1 if the participant consumes alcohol, and E = 2 if the participant does not consume alcohol); and “*ε*” is the individual-specific error. In addition, a follow-up multiple regression analysis, which used salivary serotonin concentration and trait empathy as independent variables, was conducted to reveal whether the variables of interest independently affected the dependent variable (happiness in self-positive/friend-positive condition).

Furthermore, stepwise multiple regression analysis was also used to find the most parsimonious set (independent variables: mother’s attention during childhood, father’s attention during childhood, age, sex, BMI, drinking habits) that is most effective in predicting the dependent variable (salivary serotonin concentration). In order to indicate the association between mother’s attention during childhood, trait empathy, and happiness, Pearson’s correlation coefficients were also computed.

## Results

### Sample characteristics by sex

Table 1 displays the sample characteristics by sex. In the present sample, age and BMI were higher in males than females (*p* < .01 and *p* < .05, respectively). Drinking was more prevalent among male participants than female participants. As for parents’ attention during childhood, female participants reported higher attention from their fathers than did males (*p* < .05). In terms of empathic traits, female participants had a higher fantasy score than males (*p* < .01). For happiness in the questionnaire, female participants only evaluated their happiness higher than males in the self-positive/friend positive condition (*p* < .01). No significant sex differences were evident for other psychological variables, including salivary serotonin concentration.

**Table 1:** Sample characteristics by sex for study variables.

Variables	Male	Female	*p* value
Age	19.500 (1.150)	19.036 (0.758)	< .01
BMI	20.948 (2.573)	20.252 (2.315)	< .05
Number of participants with drinking habits	21	6	
Father’s attention in childhood	4.350 (1.095)	4.714 (1.150)	< .05
Mother’s attention in childhood	5.460 (0.626)	5.420 (0.755)	.674
Salivary serotonin (ng/ml)	14.893 (9.951)	15.660 (11.330)	.629
IRI Subscales			
Perspective taking	3.054 (0.705)	3.027 (0.564)	.756
Personal distress	2.962 (0.722)	3.004 (0.782)	.687
Fantasy	3.053 (0.655)	3.304 (0.610)	< .01
Vignette			
Self-positive/absent	6.360 (0.675)	6.509 (0.615)	.094
Self-positive/friend-positive	6.610 (0.584)	6.804 (0.462)	< .01
Self-positive/friend-negative	4.810 (1.022)	4.804 (1.229)	.967
Self-neutral/absent	4.490 (0.859)	4.419 (0.856)	.551
Self-neutral/friend-positive	4.610 (1.230)	4.384 (1.179)	.174
Self-neutral/friend-negative	3.540 (0.892)	3.321 (0.861)	.071
Self-negative/absent	1.810 (0.720)	1.732 (0.657)	.412
Self-negative/friend-positive	2.460 (1.344)	2.232 (1.273)	.207
Self-negative/friend-negative	1.830 (0.932)	1.732 (0.869)	.430

Results are expressed as mean (standard deviation). These study variables were compared using Student’s *t* tests.

### Effects of the presence of friends on happiness in hypothetical situations

**Fig 1:**
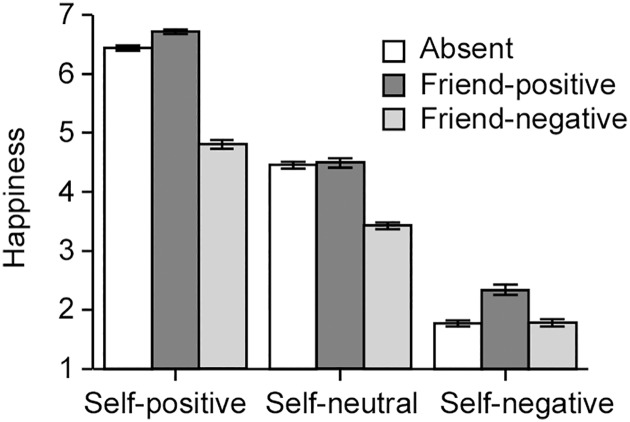
Results of the questionnaire. The bar graph shows happiness rating scores as a function of the participants’ emotional valence (positive, neutral, or negative) and the situation of a friend (absent, positive, or negative). Each column and the error bars represent means ± standard errors (*n* = 212).

Fig 1 displays the findings of the effect of the presence of friends on happiness in the vignette. A 3 (self-valence: positive, neutral, negative) × 3 (friend’s situation: absent, positive, negative) ANOVA revealed a significant main effect of self-valence on happiness rating scores, *F*(2, 1899) = 2922.191, *p* < .01, *η*^2^_p_ = .755. A multiple comparisons test indicated that the happiness rating scores in the self-positive condition (mean = 5.986 ± 0.037) were significantly higher than those in the self-neutral (4.123 ± 0.037, *p* < .01) and self-negative (1.962 ± 0.037, *p* < .01) conditions. Scores in the self-neutral condition were significantly higher than those in the negative condition (*p* < .01). This ANOVA also revealed a significant main effect of the friend’s situation on happiness rating scores, *F*(2, 1899) = 270.766, *p* < .01, *η*^2^_p_ = .222. A multiple comparisons test indicated that happiness rating scores in the friend-positive condition (4.514 ± 0.037) were significantly higher than those in the friend-absent (4.220 ± 0.037, *p* < .01) and friend-negative conditions (3.336 ± 0.037, *p* < .01). Scores in the friend-negative condition were significantly lower than those in the friend-absent condition (*p* < .01).

Furthermore, the interaction effect between self-valence and friend’s situation was also significant, *F*(4, 1899) = 48.921, *p* < .01, *η*^2^_p_ = .093. In the self-positive condition, a multiple comparisons test indicated that happiness rating scores in the friend-positive situation (6.712 ± 0.065) were significantly higher than those in the absence (6.439 ± 0.065, *p* < .01) and friend-negative (4.807 ± 0.065, *p* < .01) situations, and those in absence situation were significantly higher than those in the friend-negative situation (*p* < .01). In the self-neutral condition, happiness rating scores in the friend-negative situation (3.425 ± 0.065) were significantly lower than those in the absence (4.453 ± 0.065, *p* < .01) and friend-positive (4.491 ± 0.065, *p* < .01) situations, although there were no significant differences between absence and friend-positive situations. In the self-negative condition, happiness rating scores in the friend-positive situation (2.340 ± 0.065) were significantly higher than those in the absence (1.769 ± 0.065, *p* < .01) and friend-negative (1.778 ± 0.065, *p* < .01) situations, although there were no significant differences between absence and friend-negative situations.

### Association between trait empathy and happiness

We subsequently analyzed the correlations between the trait empathy subscales (perspective taking, personal distress, and fantasy) and rating scores of happiness in the questionnaire. As shown in Table 2, the perspective taking score was positively correlated with happiness in the self-positive/absent condition [*r*(211) = 0.179, *p* < .01; Table 2] and those in self-positive/friend-positive condition [*r*(211) = 0.175, *p* < .05; Table 2]. Furthermore, the fantasy score was positively correlated with happiness in the self-positive/friend-negative condition [*r*(212) = 0.180, *p* < .01] and those in the self-neutral/friend-negative condition [*r*(212) = 0.227, *p* < .01], and negatively correlated with the self-negative/friend-positive condition [*r*(212) = −0.137, *p* < .05]. The personal distress score was not significantly correlated with happiness in any condition.

**Table 2:** Pearson’s correlations between happiness in hypothetical situations, trait empathy subscales, and salivary serotonin concentration.

Conditions	Trait empathy subscales			Salivary Serotonin
	Perspective taking	Personal distress	Fantasy	
Self-positive/absent	0.179**	−0.044	0.001	−0.145
Self-positive/friend-positive	0.175*	−0.045	0.024	−0.194**
Self-positive/friend-negative	−0.061	−0.038	0.180**	−0.110
Self-neutral/absent	0.003	0.042	0.013	0.020
Self-neutral/friend-positive	0.016	−0.007	−0.044	0.005
Self-neutral/friend-negative	−0.041	−0.006	0.227**	0.074
Self-negative/absent	−0.062	0.066	0.031	0.094
Self-negative/friend-positive	−0.053	−0.100	−0.137*	0.133
Self-negative/friend-negative	−0.004	0.072	0.007	0.072

**p < .01,

*p < .05

### Association between salivary serotonin and trait empathy

**Fig 2:**
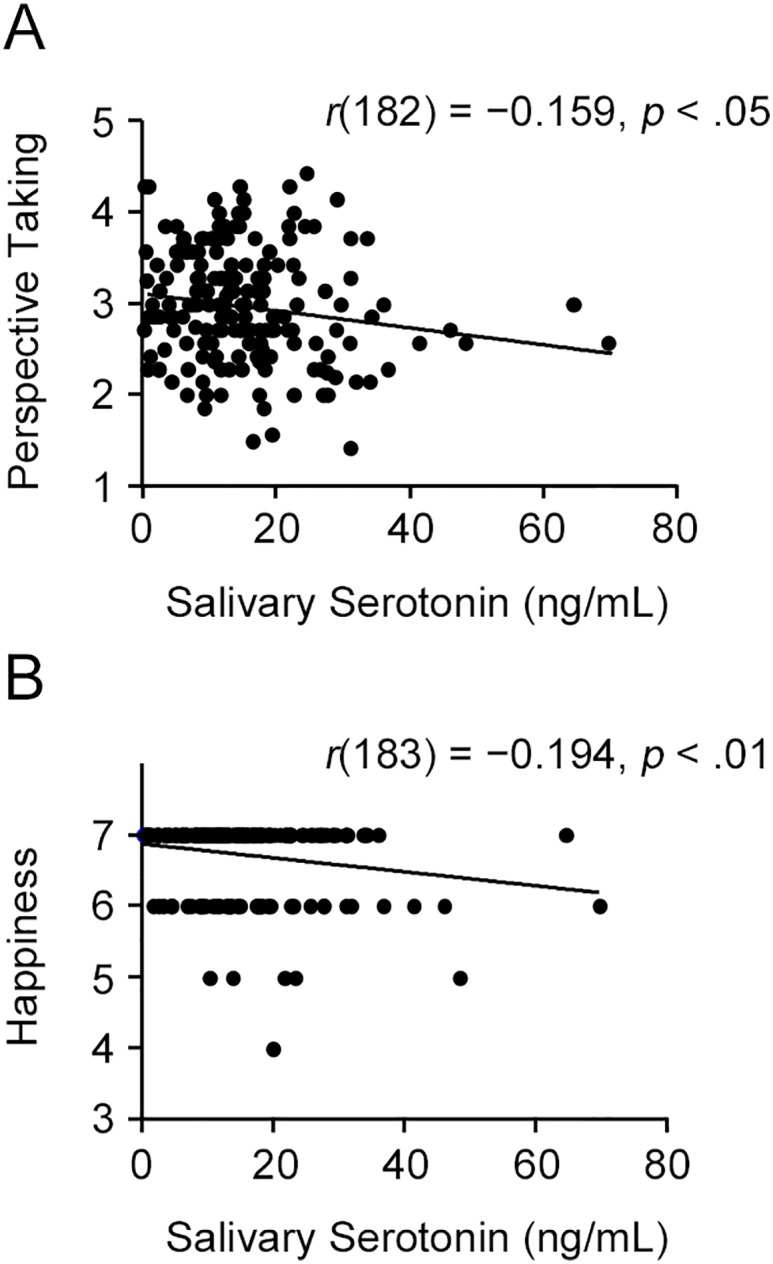
Associations between salivary serotonin levels and psychological measures. (A) The scatterplot demonstrates the negative correlation between salivary serotonin levels and perspective taking (*n* = 182). (B) The scatterplot demonstrates the negative correlation between salivary serotonin levels and happiness rating scores in the self-positive/friend-positive condition (*n* = 183).

We analyzed the correlations between salivary serotonin levels and the trait empathy subscales (perspective taking, personal distress, and fantasy). Salivary serotonin levels were negatively correlated with the perspective taking score [*r*(182) = −0.159, *p* < .05]. The scatter plot in Fig 2A shows the significant negative correlation between the salivary serotonin concentration and the perspective taking score. Correlation analysis indicated non-significant correlations between salivary serotonin levels and the fantasy score [*r*(183) = −0.092, *p* = .214], and the personal distress score [*r*(183) = 0.001, *p* = .994].

Further, we computed the correlations between salivary serotonin levels and happiness in the hypothetical life events. Salivary serotonin levels were negatively correlated with happiness in the self-positive/friend-positive condition [*r*(183) = −0.194, *p* < .01]. The scatter plot in Fig 2B shows the significant negative correlation between salivary serotonin concentration and the rating scores of happiness in the self-positive/friend-positive condition. In addition, salivary serotonin was marginally significantly correlated with happiness in the self-positive/absent [*r*(183) = −0.145, *p* = .051] and the self-negative/friend-positive [*r*(183) = 0.133, *p* = .073] conditions. None of the corresponding correlations were significant in the remaining conditions (Table 2).

### Results of the multiple regression analyses

Table 3 shows the results of the multiple regression analysis, which tested the hypothesis that salivary serotonin levels predict perspective taking scores, even after controlling for the potentially confounding variables of age, sex, BMI, and drinking habits. The model was statistically significant [*F*(5, 175) = 2.613, *p* < .05] and supported our hypothesis (*β* = −0.163, *t* = −2.231, *p* < .05).

**Table 3:** Results from the regression analysis examining the association between salivary serotonin and perspective taking.

Predictor variables	*β*	*t*	*p* value
**Salivary Serotonin**	**−0.163**	**−2.231**	**< .05**
Sex	0.003	0.045	.964
Age	−0.073	−0.928	.355
**BMI**	**0.188**	**2.520**	**< .05**
Alcohol	−0.107	−1.336	.183
*N*	182		
*Adjusted R*^*2*^	0.043		

All predictor variables were included in the regression analysis. Boldface indicates statistically significant variables. *β*: Standardized beta coefficient; BMI: Body mass index.

Table 4 shows the results of the multiple regression analysis, which tested the hypothesis that salivary serotonin levels predicted happiness in the self-positive/friend-positive condition, after controlling for the potentially confounding variables of age, sex, BMI, and drinking habits. The model was statistically significant [*F*(5, 176) = 3.905, *p* < .01] and supported our hypothesis (*β* = −0.200, *t* = −2.786, *p* < .01).

**Table 4:** Results from the regression analysis examining the association between salivary serotonin and happiness in self-positive/friend-positive condition.

Predictor variables	*β*	*t*	*p* value
**Salivary Serotonin**	**−0.200**	**−2.786**	**< .01**
Sex	0.135	1.807	.072
**Age**	**−0.178**	**−2.303**	**< .05**
BMI	−0.025	−0.341	.733
Alcohol	−0.004	−0.050	.960
*N*	183		
*Adjusted R*^*2*^	0.074		

All predictor variables were included in the regression analysis. Boldface indicates statistically significant variables. *β*: Standardized beta coefficient; BMI: Body mass index.

In addition, a follow-up multiple regression analysis, which used salivary serotonin concentration and perspective taking scores as independent variables, was conducted to reveal whether these variables independently affected the dependent variable, happiness in the self-positive/friend-positive condition. The entire model was statistically significant [*F*(2, 179) = 5.381, *p* < .01, *Adjusted R*^*2*^ = 0.046], and showed that salivary serotonin was a significant predictor (*β* = −0.170, *t* = −2.310, *p* < .05), but that the perspective taking score was not significant (*β* = 0.142, *t* = 1.931, *p* = .055).

### Stepwise regression predicting salivary serotonin levels

Finally, a stepwise multiple regression analysis was conducted to examine whether salivary serotonin levels were predicted by childhood experiences. This was done by entering age, sex, BMI, drinking habits, father’s attention during childhood, and mother’s attention during childhood in the multiple regression model. The model involving only a subset of predictor variables was statistically significant [*F*(1, 180) = 4.115, *p* < .05] and the only significant predictor was mother’s attention during childhood (*β* = −0.150, *t* = −2.029, *p* < .05). Other variables were not significant predictors of salivary serotonin levels (sex: *t* = 0.227, *p* = .821, age: *t* = 0.249, *p* = .803, BMI: *t* = 0.146, *p* = .884, drinking habits: *t* = 0.217, *p* = .828, and father’s attention: *t* = 0.991, *p* = .323).

In addition, correlation analyses revealed significant positive correlations between the rating score of mother’s attention during childhood and both the perspective taking score [*r*(211) = 0.192, *p* < .01] and happiness in the self-positive/friend-positive condition [*r*(212) = 0.177, *p* = .010].

## Discussion

We first tested whether participants’ happiness was influenced by the presence of their friends, and results supported this hypothesis. Participants rated their feelings more positively when their friends experienced positive events and more negatively when their friends experienced negative events, compared to experiencing the event alone (Fig 1). Further, correlation analysis demonstrated that happiness in the self-positive/friend-positive condition was positively correlated with the perspective taking score, and negatively correlated with salivary serotonin levels (Table 2). The multiple regression analysis indicated that salivary serotonin levels were more effective in predicting happiness in the self-positive/friend-positive condition, compared to the perspective taking score. We predicted that serotonin and perspective taking would both have a significant impact on happiness, given the theoretical basis that they share the same neural substrates associated with the sharing effect. Since only salivary serotonin levels demonstrated a significant association with happiness, this suggests that the neural substrates for the sharing effect of happiness might be more reliably reflected by salivary serotonin levels. These findings also suggest that salivary serotonin is associated with individual differences in empathic abilities and may be useful as the biomarker of empathy.

Previous studies have demonstrated that experimentally elevated serotonin attenuates the cognitive process of empathy (inferring others’ mental states) [15,16]. Because perspective taking, which is a subscale of IRI [34], was defined as the tendency to spontaneously adopt the psychological point of view of others, it reflects the cognitive process of empathy. Thus, the negative correlation between the perspective taking score and salivary serotonin levels observed may make logical sense. Furthermore, this negative correlation can also be discussed from the viewpoint of psychological well-being. Previous studies have indicated a positive correlation between perspective taking ability and psychological well-being in Japanese university students [39]. This suggests that perspective taking might help individuals control their behavior or manage their environment to achieve a better future self, through cognitive efforts toward understanding others’ perspectives. Psychological well-being may be associated with salivary serotonin levels, as indicated by a previous study that found differences in circadian rhythms between healthy individuals and patients with depression [21]. In healthy people, salivary serotonin secretion is low during the morning and high in the evening. In contrast, salivary serotonin secretion is high in the morning and low during the evening in patients with depression. Based on this finding, it is possible that psychological well-being may also be associated with circadian rhythms of salivary serotonin in university students. In the present study, saliva samples were collected between 11:00 a.m. and 1:00 p.m., during the time that salivary serotonin levels should be relatively low in healthy individuals. If perspective-taking ability were low, the peak of salivary serotonin secretion would likely be in the morning, with high salivary serotonin levels being able to be detected at this sampling point. Associations between circadian rhythms of salivary serotonin and psychological well-being in healthy university students should be further examined in the future.

Salivary serotonin levels were also negatively correlated with happiness in the self-positive/friend-positive condition. A previous neuroimaging study indicated that the sharing effect of positive emotions is associated with elevated activity in the brain’s reward system, such as the ventral striatum and orbitofrontal cortex [6]. It is known that serotonin is a fundamental modulator of emotional, motivational, and cognitive aspects of reward representation, and may have an inhibitory effect on rewarding processes [40]. An earlier pharmacological study also indicated that administration of an SSRI antidepressant reduces enjoyment in a range of activities, including social interactions, hobbies, interests, and love [41], suggesting that SSRI-induced serotonin increase inhibits motivation and pleasure. Thus, a negative correlation between salivary serotonin levels and sharing effect of happiness may make sense.

Salivary serotonin levels were not significantly correlated with the sharing effect of unhappiness (reduction of happiness by unhappy friends). Based on the results of previous studies, it is possible that increased serotonin may attenuate negative social feelings [16]; however, the present study did not demonstrate this. In the present study, it seems that the fantasy scale, which taps into respondents’ tendencies to transpose themselves imaginatively into the feelings and actions of fictitious characters in books, movies, and plays, influences the sharing effect of unhappiness (Table 2). These results seem incongruous; therefore, in the future it will be necessary to clarify the relationship between salivary serotonin levels and the sharing effect of unhappiness, using a negative emotion-induction task, such as the Cyberball paradigm [42].

Furthermore, the present study demonstrated that mothers’ attention toward their children was strongly associated with salivary serotonin levels in adulthood. Salivary serotonin levels were elevated in individuals who did not received much attention from their mothers during childhood. Correlation analysis also indicated that mothers’ attention toward their children was positively correlated with perspective taking and the sharing effect of happiness in adulthood. Previous findings have demonstrated that the environment during childhood may be critical for serotonin functions [23–26]. Thus, these results suggest that early-life stress, such as neglect from mothers, might cause alteration in central and peripheral serotonin functions and influence salivary serotonin secretion and empathic abilities even in adulthood.

We could not measure salivary serotonin levels of 30 participants due to loss of saliva samples or because levels were below the detection limit. Thus, the exclusion of samples may have affected the present findings. In order to check this, we compared the scores in perspective taking and happiness in the self-positive/friend-positive condition between the participants with and without serotonin data. Comparison of these groups revealed no statistically significant difference in happiness rating score using a Student’s t test (*t* = 0.246, *p* = .806), although the perspective taking score in the participants who did not have serotonin data (3.239 ± 0.118) tended to be higher than that in the participants who have serotonin data (3.008 ±0.046; *t* = −1.865, *p* = .064). Although we cannot deny the possibility that participants whose salivary serotonin levels were below detection limit have relatively high empathic abilities, no statistically significant differences were observed in this experiment. Thus, it is considered that the backgrounds of the excluded participants are unlikely to fall into a special category.

Our study has several limitations. First, in the vignette part of our study, we selected one occasion (When you are striving to achieve a personal goal) based on a previous experiment [36]. However, there are many occasions that can evoke happiness, such as marriage, a personal relationship, and family [36]. Thus, it is unclear whether salivary serotonin influences sharing effects of emotions in other occasions. Second, the present study found sex differences in the sharing effect of happiness. Female participants rated themselves as happier in the self-positive/friend positive condition than males. Further, the responses in several hypothetical situations, such as a marriage proposal scenario, used in the previous study [36], may also have sex-specific effects, because men are more likely than women to propose marriage. Thus, there may be interaction effects between sex and scenario in the sharing effect of emotions. Analyses that are more detailed may provide us with additional information regarding the association between salivary serotonin and empathy. Third, the sharing effect of emotions may be influenced by interpersonal closeness. A previous neuroimaging study demonstrated that positive feeling-related brain activation in the striatum during empathetic concern is enhanced by an experimental manipulation of target familiarity [42]. In the present study, participants were instructed to imagine a friend of the same gender. Thus, it is possible that the association between salivary serotonin and sharing effects may have differed according to the closeness between the pair of friends, and that there is a difference in happiness-related empathy between romantic partners and strangers. Replication of the study considering such interpersonal closeness will serve to clarify our findings in the future. Fourth, all participants of the current study were Japanese university students. Thus, it is possible that the collectivist culture may have affected the present findings, as previous studies have indicated that cultural variation can influence dispositional empathy [43]. Future studies should examine whether such cultural differences influence the association between salivary serotonin and empathic abilities.

Nevertheless, the present study shows, for the first time, that salivary serotonin is associated with individual differences in the sharing effect of happiness. Our findings indicate that salivary serotonin levels are associated with empathic abilities and increased serotonin levels might attenuate the social sharing effect of happiness. Our present results also indicate the association between childhood environment, serotonin functioning, and empathic abilities. Thus, the present findings may also be applicable to other scientific fields, including medical and educational fields.
